# Spontaneous and Drug-Induced Amyloid-Related Imaging Abnormalities: Overlaps, Divergences, and Clinical Implications Across a Continuum Between Alzheimer’s Disease and Cerebral Amyloid Angiopathy

**DOI:** 10.3390/biom16010089

**Published:** 2026-01-05

**Authors:** Marialuisa Zedde, Mattia Losa, Elena Grisafi, Davide D. Lucia, Ilaria Gandoglia, Massimo Del Sette, Matteo Pardini, Luca Roccatagliata, Rosario Pascarella, Fabrizio Piazza

**Affiliations:** 1 Stroke Unit, Neurology Unit, Azienda Unità Sanitaria Locale-IRCCS di Reggio Emilia, 42123 Reggio Emilia, Italy; 2 “The Inflammatory Cerebral Amyloid Angiopathy and Alzheimer’s Disease Biomarkers” Study Group of the SINDem (Italian Neurological Society for Dementia), 20145 Milan, Italy; 3 Department of Neuroscience, Rehabilitation, Ophthalmology, Genetics, Maternal and Child Health (DINOGMI), University of Genoa, 16132 Genoa, Italy; 4 PhD Program in Neuroscience, School of Medicine and Surgery, University of Milano-Bicocca, 20900 Monza, Italy; 5 CAA and AD Translational Research and Biomarkers Laboratory, School of Medicine and Surgery, University of Milano-Bicocca, 20900 Monza, Italy; 6 Neurology Unit, IRCCS Ospedale Policlinico San Martino, 16132 Genoa, Italy; 7 Neuroradiology Unit, Department of Health Sciences, University of Genoa, 16126 Genoa, Italy; 8 Neuroradiology Unit, Ospedale SantaAULSS5 Polesana, 45100 Rovigo, Italy

**Keywords:** cerebral amyloid angiopathy, CAA, Amyloid-related imaging abnormalities, ARIA, CAA-related inflammation, anti-amyloid therapies

## Abstract

Background: Amyloid-related imaging abnormalities (ARIA) have gained significance in the context of anti-amyloid therapies (AATs), exhibiting clinical and radiological manifestations that overlap with Cerebral Amyloid Angiopathy-related inflammation (CAA-ri). This review aims to elucidate the similarities and differences between spontaneous (sARIA) and drug-induced ARIA (dARIA). Methods: We conducted a narrative review comparing sARIA and dARIA, focusing on their underlying mechanisms, clinical presentations, and implications for diagnosis and treatment. Results: Both sARIA and dARIA are characterized by similar pathophysiological mechanisms involving amyloid deposits and neuroinflammation. Notably, ARIA can manifest as ARIA-E (edema) or ARIA-H (hemorrhage), with varying incidence rates in clinical trials. The review highlights that while sARIA occurs independently from treatment, dARIA is associated with AAT and can lead to significant symptomatic presentations. Conclusions: Understanding the continuum between sARIA and dARIA is crucial for improving diagnostic criteria, risk stratification, and therapeutic approaches. The proposed unified framework emphasizes the need for consensus in managing these conditions and advancing future research in amyloid-related diseases.

## 1. Introduction

The two primary amyloid-related brain disorders are Alzheimer’s Disease (AD) and cerebral amyloid angiopathy (CAA). The prevalence of both conditions in the general population, especially among the elderly, is notably high, with numerous pathological studies indicating that AD and CAA coexist in at least 80% of cases [1,2,3]. This significant comorbidity complicates the in vivo differentiation between the two conditions, particularly in assessing the presence or absence of one relative to the other [4]. This issue has become increasingly pertinent in recent years, especially following the initial positive outcomes from anti-amyloid therapy (AAT) trials for AD [5,6]. A key adverse event associated with AAT is the appearance of Amyloid-Related Imaging Abnormalities (ARIA), which can manifest as exudative (ARIA-E) or hemorrhagic (ARIA-H) components on brain Magnetic Resonance Imaging (MRI) [7]. ARIA is a purely neuro-radiological phenomenon, defined and standardized to identify, describe, and report this occurrence in AAT trials. The ARIA pattern closely resembles the neuroradiological aspects of the clinical-radiological presentation of CAA-related inflammation (CAA-ri) [8]. CAA-ri is a spontaneous event that occurs in patients with CAA, encompassing clinical, neuroradiological, and pathological dimensions. Typically, CAA-ri presents with acute or subacute encephalopathy and a variable neuroradiological pattern, often characterized by multilobar vasogenic edema, along with typical hemorrhagic markers of CAA, such as cerebral microbleeds (CMB) and cortical superficial siderosis (cSS). Its autoimmune pathogenesis is supported by the detection of autoantibodies against beta-amyloid in cerebrospinal fluid (CSF) [9,10,11].

The pathophysiology of ARIA and CAA is extremely similar, and while CAA-ri provides an excellent spontaneous model of ARIA-E, AAT likely accelerates the natural history of the disease, triggering CAA-related manifestations [12]. The purpose of this review is to directly compare the spontaneous and drug-induced versions of ARIA-E/CAA-ri, facilitating the development of a common terminology and unifying the two sides of the same coin [13]. We deliberately chose not to use the term “iatrogenic” ARIA to avoid ambiguity with iatrogenic CAA (iCAA), which refers specifically to post-neurosurgical CAA [14].

## 2. Methods

Given the substantial heterogeneity of the available evidence and the lack of methodologically comparable studies, we explicitly frame this work as a narrative review. Data for this Review were identified by searches—performed on 1 November 2025–of PubMed, and references from relevant articles using the search terms ((“Amyloid-Related Imaging Abnormalities” [tiab] OR “Amyloid-Related Imaging Abnormalities” [majr] OR ARIA [tiab] OR “ARIA-E” [tiab] OR “ARIA-H” [tiab] OR (“amyloid” [tiab] AND “neuroimaging” [tiab])) AND (“Cerebral Amyloid Angiopathy” [majr] OR “cerebral amyloid angiopathy” [tiab] OR “CAA-ri” [tiab] OR “CAA related inflammation” [tiab] OR “CAA” [tiab] OR (“Anti-Amyloid Treatment” [tiab] OR “Anti-Amyloid Therapy” [tiab] OR “anti-amyloid therap*” [tiab] OR “Amyloid beta-Peptides” [majr] OR “Monoclonal Antibodies” [majr] OR lecanemab [tiab] OR aducanumab [tiab] OR donanemab [tiab] OR gantenerumab [tiab] OR bapineuzumab [tiab] OR solanezumab [tiab])) AND (“2000/01/01” [PDAT]: “3000/12/31” [PDAT])]). Only original research papers published in English were reviewed. The final reference list was selected based on originality and relevance to the scope of this review.

## 3. Spontaneous and Drug-Induced ARIA: Definition and Epidemiology

The term ARIA was first introduced by Sperling et al. to describe the abnormalities seen on MRI associated with the use of Bapineuzumab, the first monoclonal antibody (mAb) tested in clinical trials for AD immunotherapy [15,16]. Since then, the interest in ARIA has considerably grown with the development of new AAT, such as Aducanumab, Donanemab, and Lecanemab, whose phase 3 studies have demonstrated statistically significant slowing of cognitive decline compared to placebo [5,6]. Based on MRI findings, two types of ARIA can be distinguished:

(I). ARIA-E (Edema/Effusion), characterized by focal regions of white matter hyperintensities (WMH), or increased signal intensities within the cortical sulci, reflecting the accumulation of protein-rich fluid within the leptomeningeal and sulcal spaces, consistent with an exudative inflammatory process.
(II). ARIA-H (Hemorrhage), defined by the appearance of CMB, cSS, or Intracerebral Hemorrhage (ICH). These findings are thought to arise from lesions related to CAA, as a result of the weakening of vessel walls and their subsequent rupture.

These events have been observed in nearly all clinical trials, although there is a significant variability in the incidence. For instance, in patients receiving high doses of Aducanumab, the incidence of ARIA-E occurs in approximately 35–36% [7]; in the TRAILBLAZER-ALZ study with Donanemab, 24.0% of treated participants developed ARIA-E, with 6.1% experiencing clinical symptomatic events [16]; and in the CLARITY AD trial with Lecanemab, the incidence of ARIA-E was reported to be 12.6% [8,17].

In comparison to ARIA triggered by to the use of AAT (drug-induced ARIA, dARIA), spontaneous ARIA (sARIA) develops independently of treatment, usually, but not always, in the context of a CAA (the so-called CAA-ri) [12,13]. In a multicenter prospective longitudinal study, Antolini et al. documented ARIA-like events in patients with CAA-ri, characterized by vasogenic edema and microhemorrhages [8]. In a case report, spontaneous ARIA has also been observed in a patient with familial AD carrying a Presenilin 1 mutation with concomitant CAA [18]. Moreover, the information that can be gleaned from the serial analysis of MRI images of patients enrolled in immunotherapy trials for AD includes sARIA in the placebo group [19]. Together, these findings support the concept that ARIA is not solely a drug-induced phenomenon but may also occur spontaneously in patients with CAA and/or AD. The exact frequency of sARIA is not yet well defined and requires further studies for precise quantification.

## 4. Biological Issues and Proposed Pathophysiology: The ARIA Paradox

**Figure 1:**
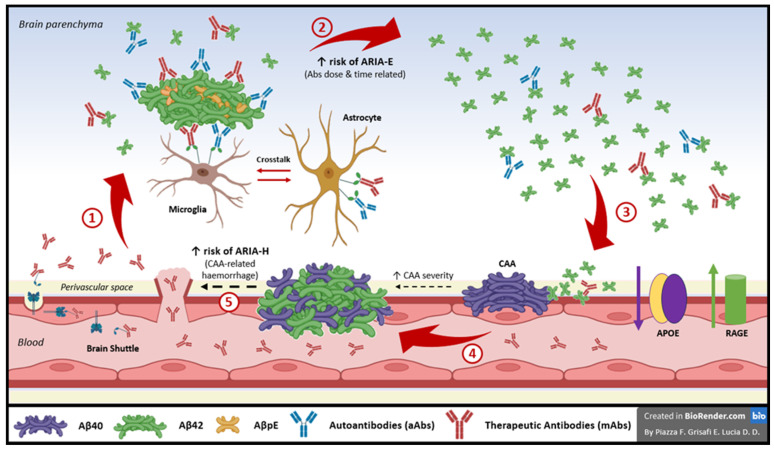
The ARIA paradox. The ARIA paradox model states that the disassembly of plaques achieved by endogenous (auto)antibodies (aAbs) in cerebral amyloid angiopathy–related inflammation (CAA-ri) and by therapeutic monoclonal antibodies (mAbs) in AAT of Alzheimer’s disease (AD) is causally linked to a paradoxical increase in CAA due to an exaggerated mobilization and relocation of Aβ from parenchymal plaques to its vascular-deposition as CAA. This leads to increased vascular perturbation and neuroinflammation (ARIA-E), which may culminate in downstream CAA-related complications (also known as ARIA-H) [11]. Red arrow 1—Therapeutic mAbs (donanemab, lecanemab) pass the blood–brain barrier (BBB) and enter the brain parenchyma through passive mechanisms (i.e., focal areas of CAA-related perturbed vascular vessels) or through active mechanisms, such as brain shuttle bispecific mAbs (trontinemab). Red arrow 2—In the brain parenchyma, mAbs targeting Aβ42 of AD plaques (lecanemab), including pyroglutamate Aβ42 (donanemab), are thought to promote microglia–astroglia reactivity, as the most accredited mechanism of action of AAT (including sTREM2 mAbs). This promotes the disassembly of Aβ plaques into circulating soluble amyloid species. In patients with (baseline) physiologically high CSF concentrations of aAbs (CAA and CAA-ri, APOE4 carriers), the sum of aAbs and mAbs leads to a rapid entry into the therapeutic window for increased risk of ARIA-E side events (red arrows 1 and 2). Red arrow 3—The antibody time- and dose-dependent increase in soluble Aβ species results in saturation of the physiological Aβ-clearance pathways, leading to an exaggerated regional and temporal redistribution of Aβ42 from parenchymal plaques to the vascular compartment (CAA). Red arrow 4—This points to a paradoxical increase in CAA, particularly in patients with pre-existing CAA and AD comorbid pathology. Both APOE4 and the “subtle” CAA-related immune and inflammatory background pathology in CAA and AD can be overstimulated, leading to an increased risk of ARIA-E and, if uncontrolled, to the downstream occurrence of new ARIA-H side events. Black arrow 5—In patients with pre-existing CAA + AD comorbid pathology, the continued dosing of mAbs or the not prompt treating with corticosteroids sustains a negative ARIA paradox loops (arrows 1–5), pointing to the transformation of MRI-undetectable ARIA, given the low sensitivity of MRI, into MRI-visible and sometimes clinically relevant ARIA manifestations [8,11,13].

A central concept in understanding the pathophysiology of ARIA is the so called “ARIA paradox” [11] (Figure 1). This phenomenon points that the removal of Aβ from parenchymal plaques, whether induced by therapeutic mAbs of AAT or driven by spontaneously occurring anti-Aβ autoantibodies (aAbs) in patients with CAA-ri, is causally linked to a paradoxical increase in CAA. This process entails an exaggerated regional and temporal redistribution of Aβ from parenchymal plaques to the vascular compartment, leading to a paradoxical increase in vascular Aβ deposition as CAA during the attempted clearance of plaque-associated Aβ from the brain parenchyma. This paradoxical exacerbation of CAA well fits with all the risk factors and downstream cascade of mechanisms though to associate with the lading of ARIA-E, including disturbances in intramural periarterial drainage (IPAD), immune- and complement-mediated neuroinflammation, and interactions with APOE genotype. When these processes remain uncontrolled, they may ultimately evolve into CAA-related complications, lately manifesting as ARIA-H on MRI [12,19]. Key open questions remain if this phenomenon requires the pre-existence of both AD and CAA comorbid pathology and what is, if any, the grade of CAA pathological severity determining the mAbs/aAbs therapeutic window for incurring in an ARIA side event. If the ARIA paradox is true, patients with isolated CAA, without AD co-pathology, as well-as AD patients without meaningful CAA co-pathology, have a reduced risk of ARIA-E [11,12].

Current research evidence suggests the mechanism of action of both therapeutic mAbs and aAbs is the disaggregation of insoluble Aβ42 of parenchymal plaques into the release of its soluble circulating species. However, when these soluble species pass the vasculature in the attempt to be eliminated through systemic clearance pathways, given the high aggregating property of Aβ, when they encounter pre-existing Aβ40 deposits of CAA, this act as a scaffold for further amyloid accumulation. This results in a progressive weakening of the CAA-compromised vessel walls, potentially worsening CAA-related immune reactivity and vessel rupture [11,20].

Post-mortem analyses from patients enrolled in early immunization trials provide evidence to support the ARIA paradox. Indeed, brains of immunized subjects showed that from 4 months to 15 years post-immunization, parenchymal Aβ42 plaques gradually diminished almost to disappearance, while CAA persisted [11,20,21]. What is particularly interesting in these patients is that CAA was no longer composed of Aβ40, but was enriched in Aβ42, which is normally present mainly in parenchymal plaques characteristic of AD. This finding aligned with the ARIA paradox model, highlighting how plaque disassembly and subsequent vascular Aβ re-distribution can generate a cycle of vascular instability that can also promote the extravasation of fluids, plasma proteins and blood cells into the surrounding tissue that explains the radiological imaging manifestation of ARIA [22].

This entire process is also closely linked to an important neuroinflammatory mechanism. Notably, in vivo studies have shown that peaks of microglial activation spatially colocalize with areas of iatrogenic ARIA-E observed during clinical trials, as well as spontaneous ARIA-E occurring in patients with CAA-ri [11]. This suggests that, beyond their role of directly disaggregating plaques, these therapeutic mAbs and endogenous spontaneous aAbs may also directly recruit and activate microglia.

Furthermore, the alterations in the vascular extracellular matrix and the exposure of damaged endothelial components can trigger a local immune response that involves microglia and astrocytes. The activation of these glial cells leads to the release of important pro-inflammatory cytokines, such as TNF-α, IL-1β, and IL-6, as well as metalloproteases, which can also act on endothelial tight junctions, exacerbating the blood–brain barrier (BBB) disruption [23,24].

This overall inflammatory response results in the increase in vascular permeability and is thought to lead to the radiological manifestation of ARIA-E, characterized by vasogenic edema. Soluble Aβ fragments, released by the disaggregation of parenchymal plaques through mAbs or spontaneously occurring aAbs, as a secondary effect, contribute to this process by continuously recruiting and activating microglia, which propagate local inflammation. This observation supports the idea that ARIA-E, which represents an early inflammatory vascular stress in both drug-induced and spontaneous ARIA, establishes a continuum that, through amyloid mobilization, vascular fragility, and general glial-mediated inflammation, may eventually lead to the more severe ARIA-H events, represented by vessel rupture and microhemorrhage. So, although split by definition, ARIA may primarily present as ARIA-E and, if not promptly managed, can potentially progress to ARIA-H [11].

The risk and severity of ARIA are also modulated by genetic and molecular factors. The APOE ε4 allele is a major known risk factor for ARIA development, with an OR = 1.9 for heterozygote carriers and OR = 5.6 in homozygotes [24]. Carriers of this allele, especially if homozygous, show an altered Aβ distribution that favors its accumulation in cerebral parenchyma and vessels, thereby increasing susceptibility to the ARIA paradox mechanism and vascular inflammation. In APOE ε4 carriers, in fact, due to high Aβ deposition, the removal pathway can trigger more severe vascular reactions, with heightened endothelial permeability and edema. Another important molecular factor involved is the microglial receptor TREM2 (Triggering Receptor Expressed on Myeloid cells 2), which acts as an immune response and Aβ clearance modulator, by influencing the balance between effective plaque removal and preservation of vascular integrity. An altered TREM2 signaling pathway can increase susceptibility to vascular damage and local inflammation, amplifying the risk of ARIA [25].

## 5. Pathological Clues: Single Case Lessons

There is no pathological definition of ARIA-E, which was first identified as a radiological manifestation in AAT clinical trials. On the other hand, CAA-ri is a well-established clinical and neuropathological entity, with diagnostic criteria subsequently validated for allowing a non-invasive diagnosis with a high degree of likelihood [26]. The active stage of CAA-ri is typically characterized from a neuropathological viewpoint by activation of microglia, T cells, and Aβ-containing multinucleated large cells surrounding CAA-positive vessel walls, signifying a spontaneous autoimmune response via Aβ autoantibodies. As a comparison, the literature includes data on several patients who experienced ARIA and died after participating in one of the immunotherapy trials [27,28,29,30].

A case from the same study of active immunization and a fatal outcome was described [27]. The patient was a 72-year-old woman enrolled in a randomized, double-blind, multiple-dose immunogenicity study of Aβ42 (AN-1792; Elan Pharmaceuticals). Four weeks after the last injection of AN-1792 (at 36 weeks after the first injection), she had the acute occurrence of drowsiness and fever. Neuroimaging showed extensive bilateral white matter hyperintense tumefactive lesions with sulcal contrast enhancement. Therapy with dexamethasone was started. The patient remained relatively stable until she died in February 2002 from a pulmonary embolism 20 months after the first injection and 12 months after the last injection. Post-mortem examination of the brain was performed, confirming a definite AD diagnosis (Braak & Braak stage V–VI). Aβ plaques were absent or sparse throughout much of the neocortex and numerous in the basal ganglia and cerebellum, which is usually a feature of relatively advanced AD. However, features of AD pathology that are not specifically associated with plaques (such as neurofibrillary tangles, neuropil threads, and CAA) were distributed relatively uniformly throughout the cerebral cortex. Some of the neocortical areas devoid of Aβ plaques contained phagocytic microglia immunoreactive for CD68 and human leukocyte antigen DR immunoreactive for both Aβ40- and Aβ42-specific antibodies. There was an infiltrate of lymphocytes in the leptomeninges, which was most dense in relation to amyloid-laden blood vessels. In addition, there was a sparse lymphocytic infiltrate in the cerebral cortex, in perivascular spaces, within the amyloid of the vessel walls, and within the parenchyma. Immunohistochemistry identified T lymphocytes (CD3+ and CD45RO+; the majority were CD4+. and very few were CD8+). In addition, there was an extensive macrophage infiltration in the white matter, which might have been responsible for the tumor-like appearance in the neuroimaging. The macrophages in the white matter were not immunostained for Aβ, perhaps because they had metabolized previously phagocytosed Aβ that was no longer immunoreactive, or because their presence was unrelated to phagocytosis of Aβ. Further cases were reported in the same period in the same trial [31]. In fact, active dosing of the study drug was suspended after the first reports of four patients who developed signs and symptoms consistent with aseptic meningoencephalitis during the preceding 3 weeks. The final number of involved patients was 18/372, all receiving AN1792. These findings were interpreted as meningoencephalitis by the authors, a consequence of the immunotherapy, but the neuroimaging findings were retrospectively found to be consistent with ARIA.

These findings were remarkably similar to features of aged PDAPP mice, which express a mutant Aβ precursor protein and normally accumulate Aβ deposits, after Aβ immunotherapy, including a low density of Aβ plaques in extensive areas of the cerebral cortex and a similar localization of Aβ to microglia [32,33,34,35,36,37]. Fc-mediated phagocytosis of Aβ by microglia in the presence of Aβ-specific antibodies was reported in an ex vivo study of plaque-laden tissue from both PDAPP mice and human AD [37]. Interestingly, the persistence of amyloid in the walls of blood vessels (CAA), despite its removal from plaques, was also observed in studies of PDAPP mice [35]. The authors proposed two hypotheses for this phenomenon:

(I) The vascular amyloid deposits may be more stable, more rapidly replenished, or less accessible, for example, to Aβ-specific antibody or phagocytes.

(II) Efflux of Aβ from the brain through perivascular drainage pathways may be stimulated by the immunotherapy and contribute to CAA [38].

Whatever the mechanism, this relative persistence of vascular Aβ may be relevant to the observation that CAA-related hemorrhage in APP transgenic mice was increased by one Aβ-specific antibody [39].

Regarding the two published cases treated with lecanemab, the pathological description provides an opportunity for a series of considerations. The first case [30] is about one patient in the extension phase of the Lecanemab trial [5] developing acute intracerebral hemorrhages after treatment with intravenous tissue plasminogen activator (t-PA) for suspected acute ischemic stroke. She was a 65-year-old patient (homozygous for the APOE ε4 allele) and the MRI after clinical deterioration showed acute right thalamocapsular infarction and innumerable multifocal cortical and subcortical hemorrhages with surrounding edema. The autopsy showed extensive multifocal intraparenchymal hemorrhages, CAA, “high-grade” AD neuropathologic changes, and diffuse histiocytic vasculitis with necrotizing vasculopathy involving amyloid deposition within (but not outside) the blood-vessel walls. In detail, multinucleated histiocytes and focal fibrinoid degeneration were described. The vascular amyloid is fragmented, and the blood-vessel wall shows infiltration by lymphocytes and histiocytes.

A second case was described [29]. A 79-year-old woman E4/E4 receiving three doses of lecanemab with progressively worsening memory impairment, seizures, and decrease in consciousness up to death. Neuroimaging revealed a vasogenic pattern of cerebral edema in the bilateral temporal, parietal, and occipital lobes with numerous microhemorrhages. Post-mortem SWI at 3 Tesla demonstrated extensive microhemorrhagic changes, most prominently in the temporal, parietal, and occipital lobes. In the pathological examination, immunohistochemistry for β-amyloid and phosphorylated tau demonstrated the presence of neuritic plaques and neurofibrillary tangles consistent with intermediate AD neuropathologic changes. While most of the plaques looked entirely typical, 21% appeared to have been “cleared”. Further, 24% of the plaques had minimal staining of amyloid deposits. These features were associated in some cases with intense immunoreactivity for IBA1 (microglia). Numerous areas of microhemorrhage were visualized histologically along with arterioles with varying degrees of fibrinoid necrosis and perivascular inflammation. The bulk of the perivascular inflammation was composed of macrophages with occasional multinucleated giant cells (stained by CD68); some T-cells were also present in the perivascular inflammatory milieu. CD68 staining of macrophages and activated microglia were present in the leptomeninges and involved some areas of parenchyma, especially near involved vessels. Areas with less prominent edema on the neuroimaging, including most of the frontal lobes, had less prominent perivascular immunoreactivity for CD68. The patient also had severe CAA. The ruptured arterioles associated with microhemorrhages contained significant β-amyloid deposits. Vascular amyloid deposition, periarterial inflammation, and microaneurysms were also frequently observed in meningeal specimens.

Recently, a pathological case–control study has been published [40]. It was a retrospective study including five aducanumab-treated participants (four males, all carrying at least one APOE ε4 allele, two harboring a PSEN1 mutation) who underwent autopsy, matched by untreated AD patients. Cumulative dosages of aducanumab ranged from 5 mg/kg to 241 mg/kg; all participants cognitively declined during treatment, and two exhibited ARIA. Reductions in [^18^F] florbetapir PET Centiloid values ranged from −6% to −81% compared with baseline. Treatment-to-death intervals ranged from 5 months to 41 months. Neuropathological analyses revealed clearance of Aβaa1–8 and Aβ42 localized to cortical layer I in treated participants, with no significant clearance in deeper cortical layers. Regions corresponding to ARIA on MRI showed microinfarcts with hemosiderin, complement activation, and CD68-positive vessel walls originating from Aβ-laden leptomeningeal and penetrating vessels. In the reported cases, the disproportionate Aβ clearance and ARIA-associated neuropathology localized to superficial cortical layers suggest a distinctive pattern of target engagement by aducanumab, likely because the antibodies have limited ability to penetrate deeper than the subarachnoid and perivascular spaces. However, the neuropathological characteristics of ARIA—such as microinfarcts, deposits of hemosiderin, and vascular inflammation—suggest again a strong connection with CAA-ri.

Another issue shared by ARIA and CAA-ri is the dual component: local inflammation with vasogenic edema and hemorrhage. In fact, similar to episodes of CAA-ri, it is well-established and supported by neuropathological data that the underlying inflammatory component is fundamental also for the pathophysiology of hemorrhages. Indeed, in 49% of ARIA-E cases, ARIA-H co-occurred and CMB may often present and accumulate over time in areas where ARIA-E is resolving or has recently resolved [41]. The other concept that can relate the inflammatory component and hemorrhages is the phenomenon of vascular remodeling (i.e., removal of Aβ in the advanced CAA stages). One of the patients pathologically examined by Kozberg et al. [28] was enrolled in the AN1792 clinical trial and had documented ARIA-E during life [27,42]. The patient was a female, aged 74 years, and the cause of death was pulmonary embolism. One occipital slab was scanned with ex vivo 7T MRI, and sections were sampled from a region with possible ARIA-H. Two hematoxylin and eosin (H&E) stained sections were screened for severe CAA vessels (Grade 4 of the Vonsattel Scale) [43]. Grade 4 vessels were identified in the cortical and leptomeningeal arterioles, and these vessels exhibited characteristics of significant vascular remodeling. In particular, the arterial walls showed fewer amyloid-β deposits than expected, probably related to the past ARIA, in addition to fibrin presence, indicative of BBB leakage. Of note, Aβ was observed in the otherwise normal-appearing wall of this vessel remote from the site of remodeling. Surrounding the vessel, there was evidence of reactive astrocytes and activated microglia, suggesting active inflammation at the site. Overall, the authors [44] demonstrated that advanced grade vessels are consistently associated with BBB leakage and perivascular inflammation. This further supports that local inflammation can be one of the driver mechanisms that lead to hemorrhagic lesions, with vascular Aβ accumulation and clearance as a potential mechanism that explains the co-occurrence of ARIA-E and ARIA-H [45].

Overall, these pathological findings show how shared inflammatory and vascular mechanisms give rise to both ARIA and CAA-ri. We next outline the clinical and neuroradiological patterns of spontaneous and drug-induced ARIA, highlighting their key similarities and differences.

## 6. Clinical and Neuroradiological Patterns in Comparison

Some considerations about the clinical manifestations may help frame the proposed differences and similarities between dARIA and sARIA. Both conditions may present with headache, cognitive or behavioral changes, seizures, or focal neurological deficits, although dARIA are predominantly asymptomatic or minimally symptomatic, and sARIA is largely associated with clinical manifestations of subacute encephalopathy. MRI acquisitions in the context of clinical trials are scheduled independently from clinical manifestations, both in the treatment and placebo arms. This likely enables the early identification of neuroradiological findings associated with a biological phenomenon before a corresponding clinical manifestation appears. For instance, an incidence of 19.8% to 24.4% of ARIA-E was reported in the treatment arm of the RCT with donanemab using the standard titration regimen, with only 5.8% symptomatic (mostly presenting with headache and confusional state) and 1.5% classified as serious ARIA-E [16]. On the other hand, in clinical practice, the schedule of neuroimaging follow-up in CAA is less codified and standardized, and it seems plausible that most of the described cases of CAA-ri have been identified by MRI studies, which were obtained in the context of acute or subacute neurological manifestations. Nevertheless, in line with what we learnt from the AAT-related counterpart, there are also reports of minimally symptomatic or asymptomatic cases of CAA-ri, and in the largest prospective series reported in the literature to date, about one-third of patients presented with a single symptom, including nonspecific subjective manifestations. The most frequent symptoms at presentation in this cohort were cognitive changes (71.7%) and focal neurologic deficits (57.5%). Relatively less frequently reported were seizures (34.5%) and headaches (22.1%) [8].

Historically, the diagnostic criteria for CAA-ri included only the radiological pattern of parenchymal exudative involvement, with or without associated leptomeningeal component; these criteria demonstrated high sensitivity (82%) and specificity (97%) [26]. However, they did not take into account the possibility of isolated leptomeningeal exudative forms, more recently described in the literature. This emerging pattern of isolated leptomeningeal involvement is another potential link between CAA-ri and ARIA [29]. In a big cohort of classical CAA-ri, only 2.7% of patients had only cSS without CMB, a radiological pattern, by contrast, more common in the recently described leptomeningeal variant of CAA-ri [8]. Frequently, these forms have a clinical onset characterized by transient focal neurological episodes (TFNEs), which have been estimated at 14.5% in a multicenter retrospective cohort study of 172 persons meeting diagnostic criteria for CAA [46]. Moreover, the description of the neuroradiological pattern of dARIA already considered not only the possibility of an isolated leptomeningeal component, but also an infratentorial localization, which was not explicitly stated in the diagnostic criteria for CAA-ri [47,48].

**Figure 2:**
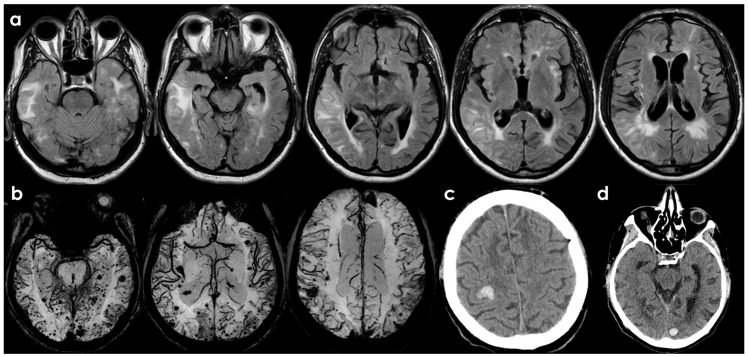
Brain MRI of a patient (male, 72 years old, past history of arterial hypertension) admitted for the occurrence of decreased alertness due to non-convulsive status epilepticus. In panel (**a**), axial Fluid Attenuated Inversion Recovery (FLAIR) slices in ascending sequences show the multilobar subcortical white matter hyperintensity with finger-like projections into the axis of cortical giri, with a tumefactive effect, simultaneous cortical swelling and sulcal effacement in the involved regions and sulcal hyperintensities. The involved regions are anterior temporal lobe on both sides, the right lateral temporal lobe extended to the lateral temporo-parietal transition and the posterior parietal lobe, and the right fronto-polar region. In panel (**b**), axial Susceptibility Weighted Imaging (SWI) images, reconstructed by using minimum Intensity Projections/MultiPlanar Reconstruction (minIP/MPR) protocol, showing an extensive burden of CMBs with few hypointense lesions > 1 cm (macrohemorrhages). In panels (**c**,**d**), non-contrast computed tomography (NCCT) of the brain at two sequential time points in the follow-up, showing acute ICH.

**Figure 3:**
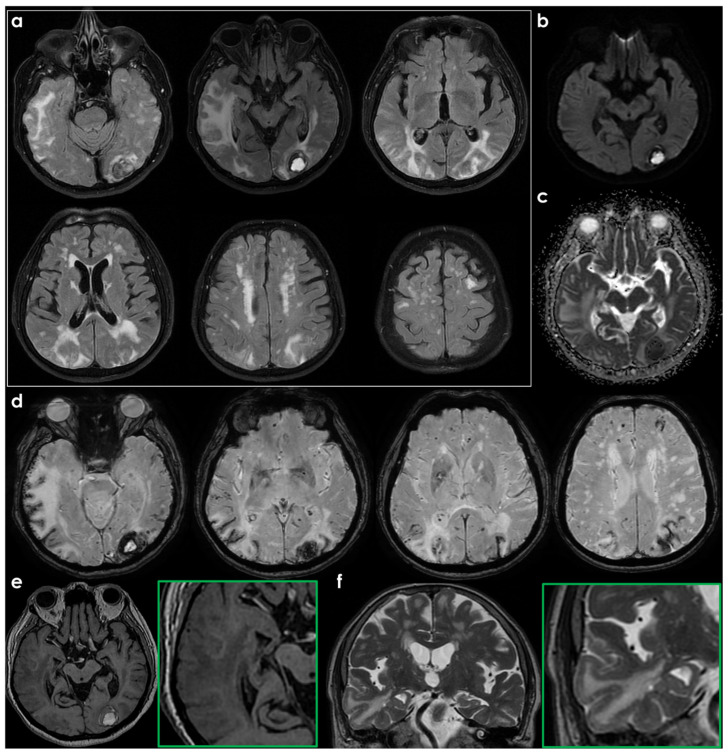
Brain MRI of a patient (male, 77 years old, past history of arterial hypertension and smoking habitus) referred to the neurologist because of a slowly progressive global impairment of the cognitive performance lasting three months. In panel (**a**), the axial FLAIR slices in a progressively ascending direction show multilobar, bilateral and mildly asymmetric white matter hyperintensities in the subcortical location, with tumefactive effect, superimposed on a pattern of chronic WMHs of presumed vascular origin in the centrum semiovale on both sides and deep lacunae. In the left occipital pole, within the above described white matter tumefactive lesions, a subacute ICH is visible as rounded hyperintese focal lesion. In panel (**b**,**c**), respectively, DWI and ADC images are proposed, highlighting the signal features supportive of vasogenic edema, corresponding to tumefactive white matter hyperintensities in the temporal lobe on both sides. In panel (**d**), SWI shows diffuse cSS, mainly corresponding with the ARIA-E topography, and in the regions the left occipital ICH. In panel (**e**,**f**), axial T1WI and coronal T2WI and magnified details, show the extension of the edema to the cortico-subcortical junction.

The neuroradiological pattern of spontaneous and drug-induced exudative forms is essentially indistinguishable in the absence of information regarding ongoing AAT (Table 1), an element relevant for understanding the pathophysiology of ARIA-E and ARIA-H [3,49,50]. In Figure 2 and Figure 3, an example of the baseline MRI of a patient with CAA-ri who presented with subacute encephalopathy is shown.

## 7. From Clinical Issues to Treatment

Spontaneous and drug-induced ARIA share overlapping mechanisms, but their management implications may differ. From a practical standpoint, the initial clinical approach (particularly in sARIA) should combine the exclusion of a broad differential diagnosis (e.g., ischemic stroke, encephalitis, or neoplastic processes), performing a dedicated MRI protocol and a CSF analysis [51,52]. Once alternative diagnoses are ruled out, early initiation of immunosuppressive therapy is generally suggested to prevent long-term neurological sequelae and mortality [8]. The non-specific manifestations, mimicking other subacute neurological conditions, and the frequent clinical comorbidities usually complicate and prolong the diagnostic pathway and the consequent management [8]. The literature is rich in case series and case reports, often retrospective and almost invariably involving patients with severe clinical presentation, in whom the propensity for treatment is immediate and usually unquestioned. The only large prospective series available also includes patients with non-severe clinical presentation, and outcome has been found to correlate not only with the execution of treatment but also with its rapidity and duration [8].

In the next sections, we will examine the main clinical challenges in managing dARIA-E and sARIA/CAA-ri, including the decision to treat, the choice of therapeutic approaches, and the related outcomes.

### 7.1. To Treat or Not to Treat?

A central clinical dilemma is whether every case of ARIA requires active treatment or whether a more conservative approach may sometimes be justified.

In the published case reports/series, with the limitation of a non-systematic and prospective patient inclusion, the diagnosis of sARIA in patients with CAA (CAA-ri) is usually made in the context of a severe symptomatic clinical episode, often characterized by rapidly progressive cognitive decline or other subacute manifestations. From the clinical point of view, these patients are usually treated when the diagnosis has been formalized, considering the severity of initial involvement, and immunosuppressive therapy is generally required. To note, as previously cited, cases of patients with a mild/asymptomatic presentation or spontaneous remitting course are reported, and in larger series, the rate of clinically mild manifestations is not negligible [8,53,54,55]. Moreover, concomitant clinical frailty and comorbidities are frequent, which globally contribute to adverse events of immunosuppression. This raises the practical question of whether treatment is warranted in all such clinical scenarios, and how aggressively it should be pursued [46,52].

In contrast, both sARIA in the placebo arms and dARIA are most frequently detected as an incidental finding on scheduled safety MRI monitoring and remain clinically silent or paucisymptomatic in the majority of cases [7]. In RCT and in real-world experiences, the suggested management strategy was conservative, with temporary suspension of the drug (in dARIA), rather than immediate immunosuppressive therapy (in both sARIA and dARIA) [16,17,56]. However, in patients presenting with significant symptoms or extensive radiological involvement (radiologically severe and/or symptomatic dARIA), corticosteroid treatment has been recommended and reported to be beneficial, reflecting therapeutic practices developed in CAA-ri [8]. Nevertheless, the clinical question remains relevant also in dARIA, given the lack of data on the prevention of secondary ARIA-H in timely treated mild-to-moderate ARIA-E and on the AAT efficacy in patients actively immunosuppressed, so reserving the treatment in selected cases [8].

### 7.2. What Treatment?

The therapeutic approach to ARIA reflects both the underlying context (spontaneous vs. drug-induced) and the severity of the presentation, particularly in dARIA, where a more standardized approach is proposed, but the trigger is theoretically removable.

As said, in dARIA, especially when of mild symptomatic or moderate asymptomatic, management is usually more conservative and primarily treated with the temporary discontinuation of the AAT itself, with close serial MRI to monitor resolution [16]. In the majority of asymptomatic cases, this approach is sufficient, as ARIA-E often resolves spontaneously over weeks. Importantly, therapeutic decisions must balance the immediate need to control ARIA against the long-term goal of continuing disease-modifying immunotherapy, a situation that has no equivalent in sARIA. It is usually possible to resume the AAT after a discussion with the patient when moderate ARIA-E is radiologically resolved (or ARIA-H stabilized), while definitive suspension is generally recommended in severe or recurrent ARIA. Severe occurring dARIA-H (defined as more than 10 microbleeds or >1 focus of cSS), also necessitates the interruption of AAT, but no information is available on the opposite strategy. To note, the definition of ‘severe’ was arbitrarily defined and not supported by biological or pathological evidence.

In symptomatic and/or severe ARIA (both spontaneous and drug-induced), high-dose corticosteroids remain the cornerstone of treatment [52]. Intravenous methylprednisolone (typically 1 g/day for 3–5 days), followed by a slow oral taper over 3–6 months, is the most widely used regimen and has demonstrated substantial clinical and radiological efficacy in observational studies. The slow tapering is suggested in sARIA, with less likely recurrence compared to corticosteroid pulse therapy, while it is not strictly recommended in dARIA [8]. Symptomatic management (e.g., antiepileptics for seizures, supportive care for encephalopathy) complements the immunosuppressive strategy.

Where there is less uncertainty is how long to maintain the steroidal treatment in sARIA, but no reliable information exists to answer the questions, if alternatives are equally valid options, and when to eventually shift to another agent for long-term management [8]. It is generally recommended, applying the experience in other immune-mediated disorders, to consider other treatments in cases of relapse or to avoid corticosteroid dependence [57]. Other steroid-sparing immunosuppressive therapies (e.g., mycophenolate mofetil, azathioprine, methotrexate) have been anecdotally employed, but it is unclear which approach is more effective in the long term, especially balancing the risk of immunosuppression. Although without published evidence, cyclophosphamide and rituximab are sometimes reserved for steroid-refractory cases, but this clinical scenario in CAA-ri should rather lead to reconsidering the diagnosis and a brain biopsy to confirm the suspicion. Further studies would compare different regimens and therapeutic attitudes, possibly harmonizing practice across centers.

### 7.3. What Are the Outcomes of Treatment?

The response to treatment likely depends on both the underlying biological context (sARIA vs. dARIA) and the therapeutic strategies employed. Furthermore, the choice of optimal outcome measures remains a matter of debate. Most observational studies on sARIA have focused on the risk of inflammatory relapse in the short term, with neuroimaging serving as the primary tool to monitor treatment response and disease recurrence. Ultimately, the main determinants of outcome probably remain the underlying hemorrhagic risk and/or disease progression with or without accompanying neurodegeneration, whose susceptibility to disease-modifying immunomodulatory treatments is still uncertain [8,47].

In sARIA, corticosteroid therapy induces a robust clinical and radiological response, with complete functional recovery seen in up to 70–80% of cases, and more than half of the patients present an apparent monophasic course [8,58]. Nonetheless, despite the absence of published cohorts with long follow-up, relapse rates remain relatively high, with up to 15–40% of patients experiencing recurrence after initial remission, justifying repeated MRI for monitoring and possibly prolonged immunosuppressive regimens [8]. Long-term functional outcomes were not fully explored in previous studies, and so, currently remain without acknowledged predictors. Some patients return in the short term close to their neurological baseline, whereas others are left with cognitive decline or focal deficits.

In dARIA, the natural course of the neuroinflammatory pattern is often more favorable, with the majority of ARIA frequently resolving within a few weeks after the AAT interruption [7,16]. Corticosteroid use in severe cases appears to accelerate the resolution of edema and improve clinical recovery. In this context, the major determinant of long-term outcome is not the ARIA episode itself: the therapeutic dilemma lies more in AAT management than in controlling inflammation per se. For this reason, memory clinic teams face the challenge of balancing ARIA risk and continuing the exposure to AAT. Future investigation should address the risk of cognitive decline or major ICH in the long-term in CAA and/or AD patients with or without spontaneous or drug-induced ARIA, providing more data on possible modifiable prognostic factors.

## 8. Discussion

As described in this review, spontaneous and drug-induced forms of ARIA/CAA-ri represent two ends of a shared pathological spectrum, both characterized by an inflammatory response to vascular Aβ deposits. We provided a table that summarizes what we comparatively described about spontaneous and drug-induced ARIA (Table 1). Although initially regarded as distinct entities, growing evidence supports a unifying framework in which CAA-ri and ARIA reflect different expressions of the same amyloid-driven, immune-mediated process [12,13].

Histopathological and imaging findings converge toward this unifying interpretation. In both cases, perivascular lymphocytic infiltration, macrophage-mediated amyloid clearance, and BBB disruption were observed. The inflammatory milieu, characterized also by activation of microglia and astroglia, was previously described in sARIA in vivo, suggesting inflammation as the pathomechanism of brain damage in these patients [11,49,50,51,52,53,54,55,56,57,58,59,60]. In CAA-ri, this process occurs spontaneously, likely triggered by a loss of immune tolerance to vascular amyloid. At the same time, in dARIA, it is pharmacologically induced by rapid Aβ mobilization during AAT [12,45]. The shared pattern of cortical–subcortical FLAIR hyperintensities, often asymmetric and posteriorly predominant, together with the coexistence of CMB and cSS, supports a common substrate. In both cases (sARIA and dARIA), the parenchymal exudative manifestations would be a facilitating element for the increase in the lesion burden of hemorrhagic markers (ARIA-H).

The role of inflammation in CAA and AD is complex and likely bidirectional [61,62,63], and the occurrence of sARIA also in the placebo arm of AD immunotherapy trials provided a useful insight into the natural history of amyloid-related disease and the coexistence of CAA and AD, even in selected patients enrolled in RCT. The overlapping immunopathology of ARIA and CAA-ri is mirrored by similar biological players. The APOE ε4 genotype appears to predispose to both ARIA and CAA-ri, underscoring a shared genetic susceptibility [64]. Naturally occurring autoantibodies directed against Aβ have been described in CAA-ri and are thought to initiate or amplify inflammation, whereas in ARIA, exogenous monoclonal antibodies provoke an analogous unwanted immune response [10]. Aβ itself is a known trigger for both local inflammation [65,66,67] and BBB leakage [62], while fibrinogen is a known trigger for microglial activation [24,68]. At the level of single-vessel segments, BBB leakage appears to occur at an earlier stage of CAA pathology than perivascular inflammation. This suggests that early BBB leakage in CAA may in fact be a driver of perivascular inflammation. The inflammation derived from innate immune responses may share mechanisms with CAA-ri and ARIA, in which inflammation has been linked to both local Aβ removal and hemorrhage [10,11,13,15,69,70].

The pathology of CAA, as compared with ex vivo MRI, provides useful information on mechanisms leading to hemorrhage in CAA beyond direct damage from Aβ deposition. In fact, recent studies examining CMBs found decreased or no Aβ as well as fibrinoid necrosis at the site of vessel rupture, causing vessel remodeling and signaling BBB leakage [71]. Kozberg et al. [45] expanded the consideration that remodeled vessels with decreased Aβ and fibrinoid necrosis are associated with hemorrhages more strongly than Aβ deposition, with perivascular inflammation occurring predominantly surrounding advanced grade vessels. This suggests the hypothesis that inflammation underlying the BBB leakage may be one of the mechanisms of CMBs formation, and that a similar pathophysiology is underlying ARIA-H due to AAT [15]. In this view, the term ARIA H could also be considered as a misnomer, since it emphasizes the neuroradiological features (i.e., microhemorrhagic MRI markers) whose pathophysiology is thought to be probably inflammatory, both at microscopic and macroscopic levels [49].

From a therapeutic standpoint, this continuum emphasizes the delicate balance between amyloid clearance and vascular stability. In a recently published multicentric cohort, 27.4% of patients with iCAA had transient inflammatory changes on MRI (cortical or parenchymal edema, sulcal hyperintensities), usually incidentally found and fully resolved on follow-up scans [72]. This high prevalence of spontaneous ARIA-E in iCAA suggests that inflammatory activity may be even more active in iatrogenic forms of CAA, pointing to a possible major pathophysiological role in iCAA progression [73]. The growing evidence about a wider role of neuroinflammation in CAA pathophysiology and CAA patients with higher hemorrhagic risk (i.e., iCAA, CAA with TFNE or with cSS) raises the possibility of a modulating effect of CAA-related hemorrhagic risk by treating inflammation [8,74,75,76,77,78]. Evidence about these therapeutic strategies in sporadic CAA and in dARIA, with the idea of preventing ARIA-H, is currently low and should be addressed by prospective studies. Recognizing these entities as manifestations of a single, dynamic process reframes the CAA continuum not as a static deposition disorder but as a dynamic immune-reactive vasculopathy. This integrated view may ultimately guide the development of targeted immunomodulatory approaches and improve understanding of how neuroinflammation shapes disease evolution at the interface between cerebrovascular and neurodegenerative pathology. With next-generation monoclonal antibodies optimized for improved blood–brain barrier delivery and reduced ARIA liability, the incidence of drug-induced ARIA is expected to decrease, while spontaneous forms of CAA-ri may correspondingly gain prominence [79,80]. Lessons learned from ARIA monitoring in clinical trials (e.g., systematic MRI surveillance, standardized grading) could inform management strategies for CAA-ri, which is currently mostly empiric and discretionary. Further studies should also explore emerging biomarkers (such as CSF and plasma inflammatory markers, and TSPO-PET) as methods to assess baseline risk and monitor therapeutic response in spontaneous and drug-induced ARIA [11,79,81,82].

## 9. Conclusions

In summary, accumulating pathological, biological, and radiological evidence indicates that spontaneous and drug-induced ARIA/CAA-ri reflect a continuum of Aβ-driven, immune-reactive vasculopathy rather than distinct entities. Recognizing this clarifies why both forms overlap in clinical presentation and imaging features and supports the need for harmonized management strategies across settings. Future work should define biomarkers capable of predicting ARIA susceptibility, clarify the long-term consequences of these events, and evaluate how immunosuppressive or preventive approaches may mitigate risk while preserving therapeutic benefit. A unified conceptual framework will ultimately improve risk stratification, monitoring, and personalized care for patients with CAA or exposed to AAT.

## Figures and Tables

**Table 1:** Comparison between spontaneous ARIA (CAA-ri spectrum) and drug-induced ARIA.

	Spontaneous ARIA (sARIA)	Drug-Induced ARIA (dARIA)
Epidemiology	Prevalence of sARIA-E in the placebo arm of RCTs was 1.7–1.9%. Real-world prevalence of sARIA or CAA-ri is unknown, while sARIA was detected in 27.4% of patients with iCAA of a multicentric cohort.	Relatively common during treatment with first-generation AAT, occurring in 10–30% of treated patients, depending on the agent, dosing, APOE ε4 status and MRI markers at baseline.
Proposed Pathophysiology	Immuno/inflammatory reaction triggered by endogenous anti-Aβ autoantibodies, leading to vascular fragility, microglial activation, and inflammatory BBB breakdown.	Triggered by therapeutic anti-Aβ monoclonal antibodies that disaggregate Aβ42 plaques, promoting downstream vascular deposition, microglial-mediated inflammation, and increased permeability in small vessels.
Neuropathological features	CAA-ri is usually characterized by perivascular inflammation with activated microglia, T-cells, and multinucleated Aβ-containing cells surrounding CAA-affected vessels.	Characterized by plaque clearance with persistent or enhanced vascular Aβ, microglial activation, perivascular macrophages, fibrinoid necrosis, and inflammatory vasculopathy.
Clinical Manifestations	CAA-ri typically presents with subacute encephalopathy, cognitive decline, or focal deficits. MRI is often performed during symptomatic episodes, though minimally symptomatic cases were also described. In the placebo arm of RCTs, most of the spontaneous ARIA-E were asymptomatic.	Most cases are asymptomatic or mildly symptomatic—detected through scheduled MRI monitoring—with occasional headache, confusion, or seizures, and only a small minority developing clinically significant ARIA-E.
Neuroimaging characteristics	CAA-ri is usually characterized by: -Unifocal or multifocal hyperintense lesions (cortico-subcortical or deep) that are asymmetric and extend to the immediately subcortical white matter -Presence CMB or cSS, frequently colocalized with ARIA-E Although not included in the CAA-ri diagnostic criteria, leptomeningeal involvement similar to the drug-induced counterpart have been described.	ARIA-E may present as parenchymal edema, sulcal effusion, or a combination of both. The imaging pattern is similar to vasogenic edema, represented by hyperintense signal on T2-FLAIR in the white matter, gray matter, or both. There may be associated local mass effect and gyral swelling. ARIA-H includes CMB, cSS (frequently colocalized with ARIA-E), or lobar ICH.
Management and therapeutic approach	Diagnosis requires exclusion of alternative causes, after which early high-dose corticosteroids with a prolonged taper should be administered to prevent neurological deterioration. Because patients may relapse, long-term immunosuppression with steroid-sparing agents are sometimes required.	Management is usually conservative, with temporary suspension of the AAT and serial MRI surveillance, as most cases resolve spontaneously without immunosuppression. Corticosteroids are reserved for symptomatic or radiologically severe ARIA, while long-term treatment decisions focus on balancing ARIA risk with the continuation of disease-modifying therapy.

## Data Availability

No new data were created or analyzed in this study.

## Abbreviations

The following abbreviations are used in this manuscript:

AAT	Anti-Amyloid Therapy
aAbs	Auto-Antibodies
AD	Alzheimer’s Disease
APOE	Apolipoprotein E
ARIA	Amyloid-Related Imaging Abnormalities
ARIA-E	Amyloid-Related Imaging Abnormalities with Edema/Effusion
ARIA-H	Amyloid-Related Imaging Abnormalities with Hemorrhages
BBB	Blood–Brain Barrier
CAA	Cerebral Amyloid Angiopathy
CMB	Cerebral Microbleeds
CSF	Cerebrospinal Fluid
cSS	Cortical superficial siderosis
iCAA	Iatrogenic Cerebral Amyloid Angiopathy
ICH	Intracerebral Hemorrhage
mAbs	Monoclonal Antibodies
TFNE	Transient Focal Neurological Episode
TREM2	Triggering Receptor Expressed on Myeloid cells 2
